# Predictive Role of Physical Activity and Health-Related Quality of Life in Police Officers’ Work Assessment

**DOI:** 10.3390/ejihpe14020020

**Published:** 2024-01-29

**Authors:** Paula Alexandrina Faria, Vanessa Santos, Luís Miguel Massuça

**Affiliations:** 1 Higher Institute of Police Sciences and Internal Security, 1300-663 Lisbon, Portugal; paffaria@psp.pt; 2 ICPOL, Higher Institute of Police Sciences and Internal Security, 1300-663 Lisbon, Portugal; vanessa.santos@ipiaget.pt; 3 First Responder Research Laboratory, University of Kentucky, Lexington, KY 40506, USA; 4 Exercise and Health Laboratory, CIPER, Faculty of Human Kinetics, University of Lisbon, 1495-751 Cruz Quebrada, Portugal; 5 CIDEFES, Lusófona University, 1749-024 Lisbon, Portugal; 6 CIFI2D, Faculty of Sport, University of Porto, 4200-450 Porto, Portugal

**Keywords:** physical activity, professional performance, quality of life, police, health

## Abstract

Police officers (POs) frequently encounter high stress and burnout risks in their demanding professional environment. This study delves into the relationship between physical activity (PA), health-related quality of life (HRQoL), and job performance among POs. A cross-sectional survey was conducted involving 1175 POs, with 691 providing complete responses. The survey included questions on biosocial and professional characteristics; the International Physical Activity Questionnaire—short form; the Short Form Health Survey version 2.0; and a qualitative job performance evaluation. The key findings highlight that vigorous PA significantly enhances job performance. About 46.2% of POs engage in vigorous PA, with a notable 73.7% participating in some form of PA weekly. This study also found that age and gender considerably impact the HRQoL, especially in mental health aspects like vitality and social functioning. Vigorous PA is linked to higher job performance ratings, especially when practised consistently. In conclusion, this research underscores the importance of vigorous PA in improving job performance among POs. We suggest that institutions prioritise facilitating environments that encourage regular PA, recognising its substantial benefits in both professional effectiveness and the overall health of POs. This study contributes to understanding the critical role of physical fitness in enhancing the occupational well-being of law enforcement personnel.

## 1. Introduction

Police officers (POs) are exposed to external and internal pressure, operating in sensitive urban areas, which can lead to high levels of stress and psychological and physical burnout [1,2,3,4].

Lagestad realised that it is essential that POs have an excellent physical aptitude to face and deal with situations that require physical strength but also an excellent psychological capacity [5]. Due to this, POs should have physical and psychological capabilities that enable them to respond to the occurrences in the best way possible.

According to the literature, frequent physical activity (PA) is associated with a better psychological condition, leading to a decrease in stress, anxiety, and depression [6]. Moreover, it is related to increased quality of life [7,8], productivity, satisfaction at work, capacity to make decisions, professional performance, and reduced absenteeism and related costs [9,10,11,12,13,14,15,16,17,18]. In this context, Steinhardt et al. observed that sedentary POs are more absent than those who are more physically active [16]. In addition, Quigley observed that most early retirements in the security forces result from low physical aptitude [14]. This is why providing conditions for POs to increase their PA level (PAL) is essential.

PA is a widely studied variable, highlighting in this context some studies such as those carried out by (i) Vatan et al., who concluded that PA had a statistically significant positive impact on resilience and workers’ efficiency [17]; (ii) Mohamed and Ghalab, who observed that PA had a statistically significant positive impact on professional performance [19]; and (iii) Burton et al., who showed that more active workers are more productive than inactive workers [11].

Against this backdrop, it is crucial to consider the broader context of PA trends, especially among the youth, as they form the future workforce in demanding professions such as policing. Recent studies have raised alarms about the increasing trend of youth inactivity and its implications. For instance, Sanz-Martín et al. highlight the growing prevalence of sedentary lifestyles among young people and the potential long-term health consequences [20]. Sanz-Martin et al. also emphasise the public health implications of this inactivity, particularly concerning occupational health and performance [21]. Furthermore, González-Valero et al. provide insights into how early lifestyle choices can impact physical and psychological well-being in high-stress careers like law enforcement [22]. This emerging evidence underscores the importance of our study, which examines the interplay of physical activity, health-related quality of life, and job performance in the context of Portuguese POs.

The literature suggests that PA and health influence professional performance [1]. Thus, we understand the importance of improving PO health by mitigating harmful effects resulting from the particularity of the PO function and the absence of a healthy lifestyle. As we have seen, this improvement reduces absenteeism and costs resulting from eventual medical treatments. It increases productivity and performance at work, being a capital gain, reducing the operational costs of the institution and improving institutional image.

Since PO activity requires physical aptitude by POs and we are not aware of studies about the impact of PA on the quality of life and professional performance of POs from the Portuguese Public Security Police (PSP), it seems relevant and appropriate to study the relation and significance of PA and health-related quality of life (HRQoL) with PO job performance. In accordance, this study aims (i) to describe the POs’ PA, HRQoL, and job performance and (ii) to evaluate the significance of PA and HRQoL attributes in predicting PO job performance. To further explore these relationships, the following hypotheses have been formulated: (H1) POs engaging in higher levels of PA will exhibit better job performance; (H2) a higher HRQoL among POs is associated with enhanced job performance; and (H3) the combined effect of high PA and good HRQoL will show a stronger correlation with superior job performance in POs than either factor alone. These hypotheses will guide the analysis and interpretation of the data, providing a structured approach to understanding the interplay between PA, HRQoL, and job performance in the context of Portuguese POs.

## 2. Materials and Methods

### 2.1. Study Design

This study is characterised as an epidemiological descriptive survey in a cross-sectional study. It was carried out by applying a questionnaire sent by institutional email and approved by the National Direction of Portuguese Public Security Police (PSP). It was applied to the POs who worked in Portugal by the civil year 2022 (operational contingent of POs of PSP). The presentation letter, shared on October 13th, 2022, through institutional email, had a hyperlink which directed those who were interested in participating to the research tool applied in this study, which included (i) the following independent variables: biosocial and professional data; the International Physical Activity Questionnaire—short form (IPAQ); and the Short Form Health Survey version 2.0 (SF-36v2). It also included (ii) as a dependent variable, the qualitative evaluation of PO job performance (last institutional assessment). From 1175 collected surveys, the ones with unfinished answers were removed from the questionnaires. A total of 691 complete/valid questionnaires were collected through a probabilistic, aleatory, and stratified sample, corresponding to an error margin of 3.66%, to a level of 95% confidence.

### 2.2. Participants

This study involved 691 POs (stature, 175.5 ± 6.3 cm; weight, 81.52 ± 11.20 kg; body mass index, 26.43 ± 3.09 kg/m^2^), observing that (i) the number of male participants (91.8%; stature, 176.3 ± 5.7 cm; weight, 82.98 ± 10.29 kg; body mass index, 26.69 ± 2.97 kg/m^2^) was superior to the number of female participants (8.2%; stature, 166.8 ± 5.2 cm; 65.30 ± 7.65 kg; body mass index, 23.49 ± 2.85 kg/m^2^); (ii) the age range with less participation was the one from 18 to 29 years old (age classes: 18–29 years, 5.4%; 30–39 years, 23.0%; 40–49 years, 37.9%; ≥50 years, 33.7%); (iii) the most represented professional category was the officers (officer, 65.6%; Chief, 17.4%; Official, 17.1%); (iv) 60.5% of the participants have worked as POs for more than 20 years; and (v) the POs from the Metropolitan Command of Lisbon are the most represented (25.5%).

### 2.3. Biosocial characteristics, PA, HRQoL, and PO Job Performance

All the members of the PSP were contacted through institutional email to take part in this study and to answer an online survey (shared on October 13, 2022, via the following link: https://docs.google.com/forms/d/e/1FAIpQLSd7ARELrMrhUseBvlmrxC2HH3STEmVrWpuzF-vxc8WTGNClmw/viewform?usp=sf_link, accessed on 13 October 2022) which included four dimensions, i.e., (i) biosocial and professional characteristics; (ii) assessment of PA; (iii) assessment of the HRQoL; and (iv) qualitative evaluation of PO job performance (institutional). There were 1175 participants, but 691 answered the survey (i.e., valid and complete answers).

Regarding biosocial and professional characteristics (initial part of the online questionnaire), several questions were set to characterise the participants, i.e., gender (male; female); age range (<29 years; 30 to 39 years, 40 to 49 years; >50 years); height (in cm) and weight (in kg); and professional context (years on duty, and professional category—officer, Chief, or Official).

The IPAQ was created to assess PA and inactivity in the European Union, in general, and in each country [23,24]. Craig et al. conducted a study in 12 countries, including Portugal, whose aim was to assess the reliability and validity of this tool. There are two versions of the IPAQ (short and long form) [25]. The short version is recommended for studies at national and international levels and generates information about the length (in minutes) and frequency (in days) of determined activities, such as walking, PA of moderate and vigorous intensity, and in sedentary activities, tasks performed. The calculations were performed, and the obtained result, according to the IPAQ [26], the PA Level (PAL) of all participants, was classified as follows: (i) Level 1 (light)—the individuals do not correspond to the criteria of categories 2 or 3 and are considered inactive individuals; (ii) Level 2 (moderate)—corresponds to the activities of intensity that reach a minimum of 600 MET-minutes/week, i.e., the individuals are in this PAL when they practice (a) 3 or more days of vigorous activity with a minimum length of 20 min a day, (b) 5 or more days of activity of moderate intensity or walk minimum 30 min/day, or (c) 5 or more days of any walk combination at a moderate or vigorous intensity; and (iii) Level 3 (high)—includes activities of intensity that reach a minimum of 3000 MET-minutes/week, observing (a) activity of vigorous intensity for a minimum of three days and accumulation of a minimum of 1500 MET-minutes/week or (b) 7 or more days of any walk combination at a moderate or vigorous intensity.

The SF-36v2 health survey is one of the most widely applied tools worldwide to measure health-related quality of life. The HRQoL [8] is one of the most studied tools, published in newspapers and scientific magazines [27]. The SF-36v2 has been translated and validated in over 30 countries, including Portugal [28]. In this study, the translated version was adapted (culturally) and validated by Ferreira [29]. The SF-36 consists of 36 short answers and assesses eight health-related dimensions that can be grouped into two components: (i) physical (physical functioning; role—physical; bodily pain; and general health) and (ii) mental (vitality; social functioning; mental health; and role—emotional). The eight dimensions consist of diverse items (2 to 10). They are scored on a type of Likert scale and, after calculating each question’s quotation, considering the recodification of some items (see Ferreira, 2000) [29], the total score of each question is obtained. Then, the raw data of the dimensions are analysed, and a higher score corresponds to a better perception of the health condition.

The PO job performance assessment was considered the last institutional evaluation, and the final score (quantitative) was converted to a qualitative scale, i.e., (i) 1 to 1.999, “insufficient”; (ii) 2 to 2.999, “sufficient”; (iii) 3 to 3.999, “good”; and (iv) 4 to 5, “very good”.

### 2.4. Statistical Analysis

To characterise the sample, descriptive statistics were analysed, i.e., measures of central tendency (mean, M) and dispersion (standard deviation, SD). In the first approach to the data, and after applying the Kolmogorov–Smirnov test, histogram observation, and Cronbach’s alpha observation for SF-36v2 (α = 0.601), the methodological option of using non-parametric tests to assess the significance of the differences in the study variables was assumed. The Chi-square test (*X*^2^) of independence was used to determine (i) if the occurrence of qualitative variables of PA, HRQoL, and evaluation of PO job performance depends on the gender of the participants, age class, professional category, and years of duty and (ii) if the occurrence of PA and HRQoL depends on the evaluation of PO job performance. The non-parametric Mann–Whitney U test was used to assess the significance of the differences (i) in the quantitative variables of PA and HRQoL between both genders (male; female) and (ii) of the variables PA and HRQoL in two qualitative contents of evaluation of PO job performance (“good” and “very good”). The non-parametric Kruskal–Wallis test was applied to assess the significance of the differences in the quantitative variables of PA and HRQoL between age class, professional categories, and years on duty. To determine the significance of the significant variables of PA and HRQoL in the probability of having “very good” (1) in the evaluation of PO job performance, logistic regression (Enter method) was applied. The Statistical Package for the Social Sciences (IBM Corp. Released 2021. IBM SPSS Statistics for Windows, Version 28.0. Armonk, NY, USA: IBM Corp) was the computer program used to analyse descriptive and inferential statistics.

## 3. Results

In this section, only the results of the descriptive analysis and the significant results of the statistical analyses carried out will be presented. However, all the results of the statistical analyses are available in the “Supplementary Material”.

### 3.1. Physical Activity (PA)

Concerning the assessment of the PA time (and energy) spent in a week at different levels of PA, it was observed that (i) vigorous PA was the one that had a superior expenditure of energy. It had 1331.13 ± 1846.59 MET-minutes/week (2.00 ± 2.05 days/week and 58.80 ± 76.70 min/week), followed by (ii) moderate PA with 945.63 ± 1074.24 MET-minutes/week (2.93 ± 2.19 days/week and 75.81 ± 88.92 min/week) and (iii) walking with 853.56 ± 927.39 MET-minutes/week (3.95 ± 2.36 days/week and 64.83 ± 84.96 min/week). On average, the energy spent in a week was 3130.33 ± 3063.31 MET-minutes/week (5.67 ± 2.15 days/week and 174.28 ± 119.64 min/week). The descriptive results are presented in Table 1.

**Figure 1:**
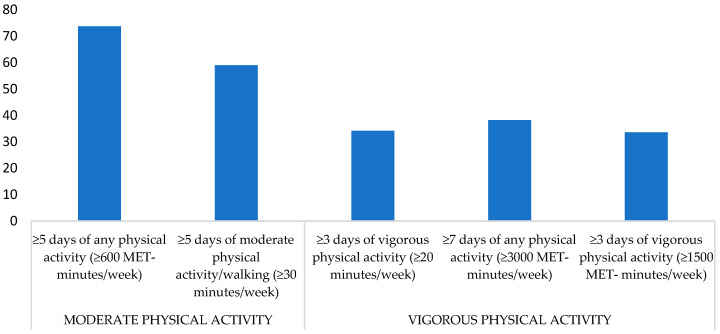
Distribution (%) in moderate and vigorous physical activity criteria of POs (*n* = 691).

Concerning the classification of the participants into categories of PA, 46.2% of the POs have a vigorous PA level (PAL), 28.2% moderate PAL, and 25.6% light PAL. Referring to the moderate PAL, 73.7% practice any PA at least for five days (≥600 MET-minutes/week), 59.0% practice at least five days of moderate/walk PA (≥30 min/day), and 34.2% practice at least three days of vigorous PA (≥20 min/day). Regarding vigorous PA, 38.2% practised seven days of any PA (≥3000 MET-minutes/week), and 33.6% practised at least three days of vigorous PA (≥1500 MET-minutes/week). The descriptive results are presented graphically in Figure 1.

**Figure 2:**
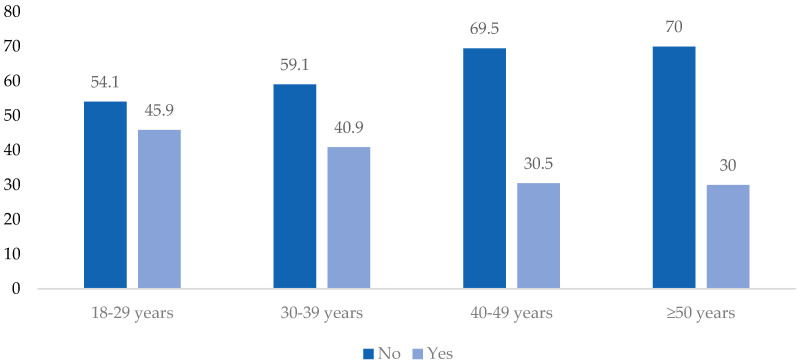
Distribution (%) in the criteria of vigorous physical activity (≥3 days of vigorous physical activity, *X*^2^(3) = 8.734, *p* = 0.033) according to age classes of POs.

It was observed that age classes have a statistically significant effect on the time spent performing PA in a week (walk (days), *p* = 0.032; vigorous activity (days), *p* = 0.013), on the weekly energy expenditure (vigorous activity, *p* = 0.021), and on the criteria of vigorous PA (≥3 days of vigorous PA, *p* = 0.033). Table 2 presents a comprehensive summary of the key results obtained from our study. It highlights the statistical analysis of the relationships between PA, HRQoL, and job performance among POs, showcasing how variables like gender, years on duty, and professional category influence these relationships. Notably, the table illustrates significant trends and correlations, clearly depicting how these factors interact in the context of our study’s objectives. The results are also presented in Figure 2.

### 3.2. Health-Related Quality of Life (HRQoL)

Regarding the HRQoL attributes, particularly in (i) the physical component, the dimension with a superior percentage is physical functioning, and the lowest is role—physical; (ii) in the mental component, the dimension with the superior percentage is social functioning, and the lowest is role—emotional. The descriptive results are presented in Table 3.

It was observed that (i) gender had a statistically significant effect on the mental component (vitality, social functioning, and mental health), and (ii) age classes had a statistically significant effect on the physical component (physical functioning; role—physical; bodily pain; and general health). The results are presented in Table 4.

### 3.3. Professional Performance Assessment

In the qualitative job performance assessment, a statistically significant effect of the weekly time spent (vigorous activity—minutes: good, 31.94 ± 58.99; very good, 59.52 ± 77.03; *U* = 7883.5, *p* = 0.025) was observed. The logistic regression revealed that vigorous PA (minutes) (*b* = 0.068, *X*^2^_Wald_ (1) = 96.735, *p* < 0.001, *OR* = 1.071) demonstrates a statistically significant effect in *Logit* on the probability of the POs being assessed in their performance as “very good”, according to the adjusted model (G^2^ (1) = 521.650, *p* < 0.001; *R*^2^_C S_ = 0.530; *R*^2^_N_ = 0.707; Table 5). The probability of a better score in the qualitative job performance assessment increases exponentially with the score of vigorous activity (minutes) (7.1% each minute), and the adjusted logistic regression model was applied to classify the sample individuals, having observed a percentage of correct classification of 97.4%.

## 4. Discussion

This research endeavours to (i) systematically delineate the nuances of physical activity (PA), health-related quality of life (HRQoL), and job performance among police officers (POs) and (ii) critically analyse the impact of PA and HRQoL on the job performance of POs.

A comprehensive literature review reveals that PA brings many benefits, including enhanced life quality, health improvement, and heightened productivity and efficiency in professional settings. Given the unique demands of law enforcement roles, a heightened PA level (PAL) is particularly beneficial for POs. This study, therefore, focuses on exploring the interplay between PA and quality of life and the implications these factors have on professional performance within a law enforcement context. Similarly, Arujunan et al. (2021) observed a significant relationship between job stress, job performance, and motivation among police officers, suggesting that enhanced physical activity levels may play a key role in modulating these aspects [30].

In the general characterisation of physical activities among participants, it was found that a significant portion of POs engage in high levels of PA. Specifically, 46.2% of the POs reported a vigorous PAL, 28.2% moderate PAL, and 25.6% light PAL. Notably, most POs (74.4%) are active, which is remarkable given that 71.6% are over 40 years old, an age group typically associated with lower PALs [31]. This finding indicates a higher level of PA among POs than the general Portuguese population, where only 27.1% exhibit a vigorous PAL, 30.3% a moderate PAL, and 42.6% a light PAL [32].

Compared with international studies, the PAL of our sample surpasses several benchmarks. This includes the analysis by Ferraz et al. [33] with military police from Cuiabá, Brazil, where 52.7% were physically active; the study by Jesus and Jesus [34] with military police from Feira de Santana, Bahia, reporting that 63.0% were active; Lorenzi’s investigation with military police from the 15th battalion of Estado de Santa Catarina, Brazil, showing that 66.0% were active [35]; and Soares et al., with only 42.0% of military police being physically active [36]. Our findings are akin to those reported by Soroka and Sawicki in their study with POs from Warsaw, Poland, where high levels of PA were observed [37]. Furthermore, a comparison with previous academic studies (dissertations) conducted at the Portuguese Police Academy reveals that, since 2014, there has been consistent PA among POs, predominantly at vigorous levels. However, the current study noted a slightly lower prevalence of vigorous PALs among POs than in previous years.

Regarding HRQoL, most participants perceived their health as good. The physical component of health, particularly physical functioning, was reported as high (92.6%), while physical performance was lower (34.9%). The mental component of health showed a higher percentage in social functioning (83.4%) but a lower percentage in emotional performance (35.7%). Generally, participants did not report their health as a limiting factor in daily activities. Nevertheless, issues in physical and mental health were noted to affect some aspects of daily activities and work. Interestingly, overall physical health, emotional problems, or pains did not significantly impact social relationships.

Finally, the institutional assessment scores were slightly higher in assessing job performance, with a remarkable 97.5% of officers rated as “very good”. This high level of institutional assessment underscores the strong correlation between PA, HRQoL, and job performance among POs.

Several key findings emerged in evaluating the biosocial attributes, PA, HRQoL, and job performance, highlighting the complex interplay between these factors.

It was noted that gender significantly influences the mental component of HRQoL, particularly affecting aspects like vitality, social functioning, and mental health. However, gender did not significantly impact the levels of PA, echoing national trends [32] and findings from the study of military police in Feira de Santana, Bahia, by Jesus and Jesus [30]. In line with our findings, Ahmada et al. identified various factors influencing job performance among police personnel, highlighting the complex interplay of physical health and job efficacy [38].

Age was found to play a significant role in PA and HRQoL. Notably, there was a decrease in the time spent engaging in vigorous PA and the energy expended in such activities as age increased. This pronounced trend affected the HRQoL, specifically influencing physical functions, physical performance, body pain, overall health, and changes in health status. These findings align with the observations made by Warr regarding institutional performance assessments [39].

Regarding professional categories within the police force, this study found no significant effect on the frequency of non-sedentary activity. However, a higher percentage of POs engaged in PA was observed in the Chief category (79.2%), followed by the Official category (74.6%) and the PO category (73.1%). Furthermore, the professional category significantly influences the HRQoL, particularly in physical function, body pain, general health, emotional performance, and mental health.

Lastly, the length of service post-Oath of Honor revealed exciting patterns. Officers with 21 to 25 years of service showed a higher percentage of light PA, while those with 0 to 5 years had the lowest. This variation in PAL according to length of service also had significant implications for the HRQoL, notably affecting physical function, performance, and body pain. Regarding the performance assessment, POs with shorter service lengths had lower “very good” assessment rates. In contrast, those with 16 to 20 years of service showed the highest “very good” performance rates, suggesting a correlation between service duration and PA.

The present study was conducted due to the need for more literature concerning the association and significance of PA and HRQoL in the institutional assessment of professional performance. Thus, we have as a limitation the inexistence of similar national studies, enabling us to conduct a comparative analysis with all the variables. In addition, it was impossible to find studies carried out with POs that analysed the relations among all the studied variables at an international level. Another limitation was that by comparing the POs’ PALs with the obtained results in international studies, we needed more studies that applied the short version of the IPAQ as a tool for measuring the PAL (i.e., there were comparisons with some studies that used the long version of the IPAQ).

While this study provides valuable insights into the relationship between PA, HRQoL, and job performance among POs, it is essential to acknowledge its limitations. Firstly, the cross-sectional nature of this study limits our ability to establish causality. Longitudinal studies would be beneficial to understand the changes over time. Secondly, the reliance on self-reported data may introduce bias, and future studies could benefit from incorporating objective measures of physical activity and performance. Additionally, this study focuses on a specific demographic, limiting the generalizability of the findings to other populations or contexts.

For future research, exploring similar relationships in different law enforcement contexts or geographical locations would be valuable to understanding cultural or environmental influences. Investigating the long-term effects of physical activity programs on POs’ health and job performance could provide deeper insights. Moreover, studies examining the role of mental health interventions alongside physical activity could offer a more holistic approach to improving POs’ well-being and job efficiency.

## 5. Conclusions

Considering the objectives and findings of this initial approach to clarify the effect of biosocial attributes, PA, and HRQoL on job performance, several vital conclusions emerge, painting a comprehensive picture of the interplay between the studied variables among POs.

This study found significant variations in physical activities, highlighted by differences among age classes in walking frequency and vigorous activity, professional categories in walking duration, and years of duty in several aspects of PA. These distinctions underscore the varied PA profiles within the police force, shaped by factors such as age, role, and career duration. Furthermore, the HRQoL assessment revealed notable disparities based on gender and age. These disparities manifested in different aspects of HRQoL, with (i) men and women differing in vitality, social function, and mental health and (ii) age impacting physical functioning, performance, body pain, general health, emotional performance, and mental health, suggesting a deep-rooted influence of these factors on officers’ overall well-being.

One of the most striking conclusions of this research is the impact of PA on job performance. This study demonstrated that the duration of vigorous activity emerged as a significant predictor of job performance assessments, reinforcing the idea that high levels of physical fitness are conducive to better job performance among POs.

In sum, this study has successfully provided an updated characterisation of PALs and HRQoL, establishing a novel link to professional performance assessment. It becomes clear that PA offers numerous benefits to POs. A notable finding is that more time engaged in vigorous PA positively impacts performance assessments. Despite generally good PALs among POs, a segment remains inactive, highlighting the need for increased PA to benefit POs and the institutions they represent. POs with higher levels of PA tend to show superior professional performance evaluation. Therefore, it is advantageous for institutions to foster an environment that encourages PA by providing exercise facilities, collaborating with gyms, and incorporating dedicated training periods into service routines, akin to practices in specialised units like Police Special Units.

## Figures and Tables

**Table 1:** Average scores (mean ± SD) on PA variables of POs (*n* = 691).

**Time spent per week**	
Walking (days)	3.95 ± 2.36
Walking (minutes)	64.83 ± 84.96
Moderate activity (days)	2.93 ± 2.19
Moderate activity (minutes)	75.81 ± 88.92
Vigorous activity (days)	2.00 ± 2.05
Vigorous activity (minutes)	58.80 ± 76.70
Seated (hours)	1.15 ± 1.43
Total (days)	5.67 ± 2.15
Total (minutes/week)	174.28 ± 119.64
**Weekly energy expenditure**	
Walking (MET-minutes/week)	853.56 ± 927.39
Moderate activity (MET-minutes/week)	945.63 ± 1074.24
Vigorous activity (MET-minutes/week)	1331.13 ± 1846.59
Total (MET-minutes/week)	3130.32 ± 3063.31

**Table 2:** Average scores of significant physical activity variables according to age classes of POs.

Time Spent per Week	Age Classes	Mean ± SD	Kruskal–Wallis Test
Walking (days)	18–29 years	4.38 ± 2.46	$X_{KW}^{2}$(3) = 8.809 *p* = 0.032
	30–39 years	4.01 ± 2.26	
	40–49 years	3.62 ± 2.39	
	≥50 years	4.20 ± 2.35	
Vigorous activity (days)	18–29 years	2.62 ± 2.16	$X_{KW}^{2}$(3) = 10.738 *p* = 0.013
	30–39 years	2.33 ± 2.16	
	40–49 years	1.87 ± 1.93	
	≥50 years	1.81 ± 2.04	
Vigorous activity (MET-minutes/week)	18–29 years	1945.95 ± 2133.30	$X_{KW}^{2}$(3) = 9.746 *p* = 0.021
	30–39 years	1503.95 ± 1845.61	
	40–49 years	1281.22 ± 1909.45	
	≥50 years	1171.67 ± 1704.32	

**Table 3:** Average scores (mean ± SD) of the physical and mental components of HRQoL of POs.

**Physical Component**	
Physical functioning (%)	92.55 ± 11.29
Role-physical (%)	34.86 ± 17.71
Bodily pain (%)	76.64 ± 19.03
General health (%)	50.01 ± 10.43
**Mental Component**	
Vitality (%)	49.66 ± 13.43
Social functioning (%)	83.44 ± 17.77
Role-emotional (%)	35.67 ± 19.59
Mental health (%)	40.25 ± 13.08

**Table 4:** Average scores of significant HRQoL variables according to age classes and gender of POs (*n* = 691).

**Physical Component**	**Age Classes**	**M ± SD**	**Kruskal–Wallis Test**
Physical functioning (%)	18–29 years	96.22 ± 12.07	$X_{KW}^{2}$(3) = 92.361 *p* < 0.001
	30–39 years	96.48 ± 7.67	
	40–49 years	93.00 ± 10.60	
	≥50 years	88.78 ± 12.76	
Role-physical (%)	18–29 years	33.78 ± 19.42	$X_{KW}^{2}$(3) = 7.930 *p* = 0.047
	30–39 years	32.52 ± 17.13	
	40–49 years	34.56 ± 17.35	
	≥50 years	36.97 ± 18.09	
Bodily pain (%)	18–29 years	84.28 ± 18.18	$X_{KW}^{2}$(3) = 33.821 *p* < 0.001
	30–39 years	81.80 ± 16.53	
	40–49 years	76.59 ± 18.74	
	≥50 years	71.97 ± 19.87	
General health (%)	18–29 years	49.92 ± 10.11	$X_{KW}^{2}$(3) = 9.327 *p* = 0.025
	30–39 years	48.42 ± 10.47	
	40–49 years	49.73 ± 10.54	
	≥50 years	51.43 ± 10.22	
**Mental Component**	**Gender**	**M ± SD**	**Mann–Whitney *U* Test**
Vitality (%)	Male	49.26 ± 13.34	*U* = 21,985.0 *p* = 0.006
	Female	54.02 ± 13.71	
Social functioning (%)	Male	83.99 ± 17.75	*U* = 13,567.0 *p* = 0.001
	Female	77.37 ± 17.06	
Mental health (%)	Male	39.93 ± 12.91	*U* = 20,912.0 *p* = 0.048
	Female	43.80 ± 14.49	

**Table 5:** Coefficient Logit of the model’s logistic regression from variable “qualitative job performance” regarding the significant PA variable.

Model	*B*	S.E.	*X* ^2^_Wald_	df	Sig.	Exp(*B*)	I.C. 95%	
							Lower	Upper
Vigorous activity ^A^	0.068	0.007	96.735	1	<0.001	1.071	1.056	1.086

Key: ^A^, in minutes.

## Data Availability

The data presented in this study are available upon reasonable request from the corresponding author. The data are not publicly available due to privacy and ethical restrictions.

## Supplementary Materials

The following supporting information can be downloaded at https://www.mdpi.com/article/10.3390/ejihpe14020020/s1, Table S1. Distribution (*n* (%)) and average values (M ± SD) of studied PA and HRQoL variables according to gender (male, female) of POs; Table S2. Distribution (*n* (%)) and average values (M ± SD) of the studied PA and HRQoL variables according to age classes (18–29, 30–39, 40–49, ≥50 years old) of POs; Table S3. Distribution (*n* (%)) and average values (M ± SD) of the studied PA and HRQoL variables according to the professional category (officers, Chief, Official) of POs; Table S4. Distribution (*n* (%)) and mean values (M ± SD) of the studied PA and HRQoL variables according to the years on duty of POs; Table S5. Distribution (*n* (%)) and average values (M ± SD) of PA and HRQoL variables according to the qualitative job performance assessment (institutional) of POs.
