# Supplementary material for: Predictive Role of Physical Activity and Health-Related Quality of Life in Police Officers’ Work Assessment
Source: Eur J Investig Health Psychol Educ. 2024 Jan 29;14(2):299–310. doi: 10.3390/ejihpe14020020 (PMC10887996; doi:10.3390/ejihpe14020020)
Supplement: Supplementary file 1 [file ejihpe-14-00020-s001.zip › ejihpe-2769268-supplementary.pdf]

Supplementary Files

Table S1. Distribution (n (%)) and average values (M ± SD) of studied PA and HRQoL variables according to gender (male, female) of POs.

Variables		Male	Female	Statistic [F]
Physical activity				
Time spent per week				
	Walking (days)	3.94 ± 2.37	4.07 ± 2.31	$U = 18672.5$, $p = 0.672$
	Walking (minutes)	64.71 ± 85.37	66.16 ± 81.03	$U = 17370.5$, $p = 0.624$
	Moderate activity (days)	2.90 ± 2.19	3.37 ± 2.20	$U = 20305.0$, $p = 0.117$
	Moderate activity (minutes)	75.04 ± 90.08	84.39 ± 74.90	$U = 20270.0$, $p = 0.123$
	Vigorous activity (days)	1.98 ± 2.04	2.12 ± 2.13	$U = 18592.0$, $p = 0.710$
	Vigorous activity (minutes)	58.53 ± 76.54	61.75 ± 79.09	$U = 18338.5$, $p = 0.849$
	Seated (horas)	1.14 ± 1.43	1.17 ± 1.37	$U = 17411.5$, $p = 0.649$
	Total (days)	5.65 ± 2.16	5.79 ± 2.09	$U = 18541.0$, $p = 0.705$
	Total (minutes/week)	172.99 ± 117.57	188.61 ± 141.01	$U = 18904.0$, $p = 0.563$
Weekly energy expenditure (MET- minutes/week)				
	Walking	847.04 ± 914.70	926.08 ± 1064.90	$U = 18181.0$, $p = 0.938$
	Moderate activity	909.51 ± 1021.12	1347.37 ± 1501.19	$U = 20415.0$, $p = 0.103$
	Vigorous activity	1312.95 ± 1807.00	1533.33 ± 2249.59	$U = 18438.0$, $p = 0.794$
	Total	3069.50 ± 2979.77	3806.79 ± 3840.59	$U = 19401.5$, p = 0.356
Vigorous physical activity criteria				
≥3 days of vigorous physical activity [A]	No	421 (66.4)	38 (66.7)	$X^2(1) = 0.002$, $p = 0.968$
	Yes	213 (33.6)	19 (33.3)	
≥7 days of any physical activity [B]	No	398 (62.8)	29 (50.9)	$X^2(1) = 3.136$, $p = 0.077$
	Yes	236 (37.2)	28 (49.1)	
Moderate physical activity criteria				
≥3 days of vigorous physical activity [C]	No	417 (65.8)	38 (66.7)	$X^2(1) = 0.019$, $p = 0.892$
	Yes	217 (34.2)	19 (33.3)	
≥5 days of moderate physical activity/walking [D]	No	261 (41.2)	22 (38.6)	$X^2(1) = 0.143$, $p = 0.705$
	Yes	373 (58.8)	35 (61.4)	
≥5 days of any physical activity [E]	No	166 (26.2)	16 (28.1)	$X^2(1) = 0.096$, $p = 0.757$
	Yes	468 (73.8)	41 (71.9)	
Categories of physical activity				
	Light	161 (25.4)	16 (28.1)	
	Moderate	186 (29.3)	9 (15.8)	$X^2(1) = 4.887$, $p = 0.087$
	Vigorous	287 (45.3)	32 (56.1)	
**Health- related quality of life**				
Physical component				
	Physical functioning (%)	92.74 ± 11.14	90.47 ± 12.78	$U = 16123.5$, $p = 0.163$
	Role-physical (%)	34.66 ± 17.53	37.11 ± 19.59	$U = 19236.0$, $p = 0.399$
	Bodily pain (%)	76.80 ± 18.86	74.88 ± 20.96	$U = 17388.0$, $p = 0.632$
	General health (%)	50.04 ± 10.31	49.74 ± 11.80	$U = 17933.5$, $p = 0.925$
Mental component				
	Vitality (%)	49.26 ± 13.34	54.02 ± 13.71	$U = 21985.0$, $p = 0.006$
	Social functioning (%)	83.99 ± 17.75	77.37 ± 17.06	$U = 13567.0$, $p = 0.001$
	Role-emotional (%)	35.47 ± 19.54	37.89 ± 20.12	$U = 19630.0$, $p = 0.251$
	Mental health (%)	39.93 ± 12.91	43.80 ± 14.49	$U = 20912.0$, $p = 0.048$

Key:
[A], ≥1500 MET- minutes/week
[B], ≥3000 MET- minutes/week
[C], ≥20 minutes/day
[D], ≥30 minutes/day
[E], ≥600 MET- minutes/week
[F], X^2 Test or Mann-Whitney U Test.

Table S2. Distribution (n (%)) and average values (M ± SD) of the studied PA and HRQoL variables according to age classes (18-29, 30-39, 40-49, ≥50 years old) of POs.

Variables		Age classes				Statistic [F]
		18-29 years	30-39 years	40-49 years	≥50 years	
**Physical activity**						
Time spent per week						
Walking (days)		4.38 ± 2.46	4.01 ± 2.26	3.62 ± 2.39	4.20 ± 2.35	$X^2_{KW}(3) = 8.809$, $p = 0.032$
Walking (minutes)		55.14 ± 65.47	68.32 ± 102.25	59.81 ± 67.98	69.62 ± 91.71	$X^2_{KW}(3) = 6.213$, $p = 0.102$
Moderate activity (days)		2.92 ± 2.29	2.90 ± 2.14	2.76 ± 2.11	3.16 ± 2.29	$X^2_{KW}(3) = 2.953$, $p = 0.399$
Moderate activity (minutes)		68.65 ± 91.94	65.89 ± 59.15	72.82 ± 88.32	87.08 ± 104.08	$X^2_{KW}(3) = 3.702$, $p = 0.296$
Vigorous activity (days)		2.62 ± 2.16	2.33 ± 2.16	1.87 ± 1.93	1.81 ± 2.04	$X^2_{KW}(3) = 10.738$, $p = 0.013$
Vigorous activity (minutes)		73.78 ± 76.56	61.38 ± 81.79	59.52 ± 80.41	53.85 ± 68.50	$X^2_{KW}(3) = 4.587$, $p = 0.205$
Seated (horas)		0.99 ± 1.10	1.21 ± 1.71	1.06 ± 1.15	1.23 ± 1.53	$X^2_{KW}(3) = 6.456$, $p = 0.091$
Total (days)		5.84 ± 1.92	5.84 ± 1.99	5.44 ± 2.41	5.78 ± 1.97	$X^2_{KW}(3) = 1.577$, $p = 0.665$
Total (minutes/week)		174.86 ± 137.27	172.57 ± 111.22	170.07 ± 124.38	180.09 ± 117.31	$X^2_{KW}(3) = 1.485$, $p = 0.686$
Weekly energy expenditure (MET- minutes/week)						
Walking		861.12 ± 1014.19	883.55 ± 990.78	803.59 ± 967.73	888.10 ± 819.51	$X^2_{KW}(3) = 6.393$, $p = 0.094$
Moderate activity		929.73 ± 1030.53	939.35 ± 1058.17	900.14 ± 1085.12	1003.61 ± 1083.65	$X^2_{KW}(3) = 2.139$, $p = 0.544$
Vigorous activity		1945.95 ± 2133.30	1503.95 ± 1845.61	1281.22 ± 1909.45	1171.67 ± 1704.32	$X^2_{KW}(3) = 9.746$, $p = 0.021$
Total		3736.79 ± 3519.54	3326.84 ± 3010.40	2984.95 ± 3198.14	3063.38 ± 2862.66	$X^2_{KW}(3) = 3.834$, $p = 0.280$
Vigorous physical activity criteria						
≥3 days of vigorous physical activity [A]	No	20 (54.1)	94 (59.1)	182 (69.5)	163 (70.0)	$X^2(3) = 8.734$, $p = 0.033$
	Yes	17 (45.9)	65 (40.9)	80 (30.5)	70 (30.0)	
≥7 days of any physical activity [B]	No	21 (56.8)	93 (58.5)	169 (64.5)	144 (61.8)	$X^2(3) = 1.948$, $p = 0.583$
	Yes	16 (43.2)	66 (41.5)	93 (35.5)	89 (38.2)	
Moderate physical activity criteria						
≥3 days of vigorous physical activity [C]	No	21 (56.8)	94 (59.1)	178 (67.9)	162 (69.5)	$X^2(3) = 6.473$, $p = 0.091$
	Yes	16 (43.2)	65 (40.9)	84 (32.1)	71 (30.5)	
≥5 days of moderate physical activity/walking [D]	No	13 (35.1)	65 (40.9)	115 (43.9)	90 (38.6)	$X^2(3) = 1.976$, $p = 0.577$
	Yes	24 (64.9)	94 (59.1)	147 (56.1)	143 (61.4)	
≥5 days of any physical activity [E]	No	10 (27.0)	36 (22.6)	73 (27.9)	63 (27.0)	$X^2(3) = 1.502$, $p = 0.682$
	Yes	27 (73.0)	123 (77.4)	189 (72.1)	170 (73.0)	
Categories of physical activity						
Light		8 (21.6)	36 (22.6)	71 (27.1)	62 (26.6)	
Moderate		9 (24.3)	42 (26.4)	76 (29.0)	68 (29.2)	$X^2(6) = 3.383$, $p = 0.759$
Vigorous		20 (54.1)	81 (50.9)	115 (43.9)	103 (44.2)	
**Health- related quality of life**						
Physical component						
Physical functioning (%)		96.22 ± 12.07	96.48 ± 7.67	93.00 ± 10.60	88.78 ± 12.76	$X^2_{KW}(3) = 92.361$, $p < 0.001$
Role-physical (%)		33.78 ± 19.42	32.52 ± 17.13	34.56 ± 17.35	36.97 ± 18.09	$X^2_{KW}(3) = 7.930$, $p = 0.047$
Bodily pain (%)		84.28 ± 18.18	81.80 ± 16.53	76.59 ± 18.74	71.97 ± 19.87	$X^2_{KW}(3) = 33.821$, $p < 0.001$
General health (%)		49.92 ± 10.11	48.42 ± 10.47	49.73 ± 10.54	51.43 ± 10.22	$X^2_{KW}(3) = 9.327$, $p = 0.025$
Mental component						
Vitality (%)		48.20 ± 12.75	49.21 ± 13.74	49.24 ± 12.87	50.66 ± 13.94	$X^2_{KW}(3) = 2.327$, $p = 0.507$
Social functioning (%)		82.97 ± 17.77	84.03 ± 17.25	83.89 ± 16.86	82.62 ± 19.15	$X^2_{KW}(3) = 0.048$, $p = 0.997$
Role-emotional (%)		37.30 ± 23.01	33.88 ± 18.78	35.60 ± 19.36	36.71 ± 19.84	$X^2_{KW}(3) = 2.366$, $p = 0.500$
Mental health (%)		37.39 ± 10.49	38.41 ± 11.48	40.56 ± 13.01	41.60 ± 14.36	$X^2_{KW}(3) = 4.083$, $p = 0.253$

Key:[A], ≥1500 MET- minutes/week; [B], ≥3000 MET- minutes/week; [C], ≥20 minutes/day; [D], ≥30 minutes/day; [E], ≥600 MET- minutes/week; [F], X^2 Test or Kruskal-Wallis Test.

Table S3. Distribution (n (%)) and average values (M ± SD) of the studied PA and HRQoL variables according to the professional category (Officers, Chief, Official) of PO.

Variables			PO Professional Category			Statistic [F]
			Officers	Chief	Official	
**Physical activity**						
Time spent per week						
		Walking (days)	3.87 ± 2.34	4.35 ± 2.43	3.84 ± 2.35	$X^2_{KW}(2) = 4.657$, $p = 0.097$
		Walking (minutes)	67.51 ± 87.40	67.83 ± 95.09	51.48 ± 60.38	$X^2_{KW}(2) = 6.122$, $p = 0.047$
		Moderate activity (days)	2.92 ± 2.18	3.06 ± 2.27	2.86 ± 2.16	$X^2_{KW}(2) = 0.388$, $p = 0.824$
		Moderate activity (minutes)	79.79 ± 95.10	73.71 ± 87.04	62.66 ± 61.17	$X^2_{KW}(2) = 1.394$, $p = 0.498$
		Vigorous activity (days)	1.94 ± 2.04	2.24 ± 2.25	1.97 ± 1.82	$X^2_{KW}(2) = 1.692$, $p = 0.429$
		Vigorous activity (minutes)	59.66 ± 81.45	56.87 ± 73.68	57.47 ± 59.48	$X^2_{KW}(2) = 1.206$, $p = 0.547$
		Seated (horas)	1.19 ± 1.47	1.20 ± 1.60	0.92 ± 1.02	$X^2_{KW}(2) = 5.717$, $p = 0.057$
		Total (days)	5.59 ± 2.20	5.91 ± 2.05	5.71 ± 2.06	$X^2_{KW}(2) = 3.136$, $p = 0.208$
		Total (minutes/week)	177.97 ± 121.74	172.27 ± 123.45	162.15 ± 107.02	$X^2_{KW}(2) = 1.265$, $p = 0.531$
Weekly energy expenditure (MET- minutes/week)						
		Walking	857.07 ± 952.49	978.56 ± 975.17	713.00 ± 751.02	$X^2_{KW}(2) = 4.506$, $p = 0.105$
		Moderate activity	960.64 ± 1080.73	980.17 ± 1155.68	852.88 ± 961.37	$X^2_{KW}(2) = 0.378$, $p = 0.828$
		Vigorous activity	1298.01 ± 1820.64	1526.60 ± 2156.97	1259.46 ± 1588.51	$X^2_{KW}(2) = 0.829$, $p = 0.661$
		Total	3115.73 ± 3038.21	3485.33 ± 3542.92	2825.33 ± 2586.40	$X^2_{KW}(2) = 0.744$, $p = 0.689$
Vigorous physical activity criteria						
	≥3 days of vigorous physical activity [A]	No	305 (67.3)	76 (63.3)	78 (66.1)	$X^2(2) = 0.686$, $p = 0.710$
		Yes	148 (32.7)	44 (36.7)	40 (33.9)	
	≥7 days of any physical activity [B]	No	278 (61.4)	72 (60.0)	77 (65.3)	$X^2(2) = 0.797$, $p = 0.671$
		Yes	175 (38.6)	48 (40.0)	41 (34.7)	
Moderate physical activity criteria						
	≥3 days of vigorous physical activity [C]	No	304 (67.1)	74 (61.7)	77 (65.3)	$X^2(2) = 1.271$, $p = 0.530$
		Yes	149 (32.9)	46 (38.3)	41 (34.7)	
	≥5 days of moderate physical activity/walking [D]	No	192 (42.4)	43 (35.8)	48 (40.7)	$X^2(2) = 1.688$, $p = 0.430$
		Yes	261 (57.6)	77 (64.2)	70 (59.3)	
	≥5 days of any physical activity [E]	No	126 (27.8)	25 (20.8)	31 (26.3)	$X^2(2) = 2.384$, $p = 0.304$
		Yes	327 (72.2)	95 (79.2)	87 (73.7)	
Categories of physical activity						
		Light	122 (26.9)	25 (20.8)	30 (25.4)	$X^2(4) = 2.075$, $p = 0.722$
		Moderate	124 (27.4)	38 (31.7)	33 (28.0)	
		Vigorous	207 (45.7)	57 (47.5)	55 (46.6)	
**Health- related quality of life**						
Physical component						
		Physical functioning (%)	91.82 ± 12.33	92.42 ± 9.53	95.51 ± 7.80	$X^2_{KW}(2) = 11.883$, $p = 0.003$
		Role-physical (%)	35.82 ± 18.36	34.04 ± 15.97	32.03 ± 17.51	$X^2_{KW}(2) = 4.657$, $p = 0.097$
		Bodily pain (%)	76.07 ± 19.56	73.99 ± 17.56	81.57 ± 17.62	$X^2_{KW}(2) = 11.920$, $p = 0.003$
		General health (%)	50.85 ± 10.53	49.53 ± 11.07	47.28 ± 8.84	$X^2_{KW}(2) = 10.840$, $p = 0.004$
Mental component						
		Vitality (%)	50.49 ± 14.00	48.30 ± 12.75	47.85 ± 11.54	$X^2_{KW}(2) = 5.197$, $p = 0.074$
		Social functioning (%)	82.23 ± 18.44	84.83 ± 16.70	86.69 ± 15.75	$X^2_{KW}(2) = 5.869$, $p = 0.053$
		Role-emotional (%)	37.04 ± 20.57	34.61 ± 17.80	31.47 ± 16.74	$X^2_{KW}(2) = 6.174$, $p = 0.046$
		Mental health (%)	41.10 ± 13.48	39.83 ± 13.19	37.37 ± 10.92	$X^2_{KW}(2) = 6.342$, $p = 0.042$

Key: [A], ≥1500 MET- minutes/week; [B], ≥3000 MET- minutes/week; [C], ≥20 minutes/day; [D], ≥30 minutes/day; [E], ≥600 MET- minutes/week; [F], X^2 Test or Kruskal-Wallis Test.

Table S4. Distribution (n (%)) and mean values (M ± SD) of the variables studied PA and HRQoL variables according to the years on duty of POs.

Key: [A], ≥1500 MET- minutes/week; [B], ≥3000 MET- minutes/week; [C], ≥20 minutes/day; [D], ≥30 minutes/day; [E], ≥600 MET- minutes/week; [F], X^2 Test or Kruskal-Wallis Test.

Variables		Years on Duty							Statistics [F]
		0 - 5 years	6 - 10 years	11 - 15 years	16 - 20 years	21 - 25 years	26 - 30 years	≥31 years	
**Physical activity**									
Time spent per week									
	Walking (days)	4.47 ± 2.43	3.93 ± 2.33	3.94 ± 2.26	3.74 ± 2.30	3,54 ± 2.53	3.88 ± 2.34	4.44 ± 2.22	$X^2_{KW}(6) = 12.660$, $p = 0.049$
	Walking (minutes)	61.51 ± 68.71	66.56 ± 88.39	72.95 ± 122.91	68.22 ± 68.30	54,43 ± 72.86	58.52 ± 47.03	75.57 ± 102.37	$X^2_{KW}(6) = 16.734$, $p = 0.010$
	Moderate activity (days)	3.07 ± 2.16	2.85 ± 2.35	2.72 ± 1.95	3.14 ± 2.19	2.72 ± 2.17	2.80 ± 2.20	3.36 ± 2.28	$X^2_{KW}(6) = 8.199$, $p = 0.224$
	Moderate activity (minutes)	65.58 ± 81.14	56.13 ± 59.96	68.52 ± 58.13	80.68 ± 75.02	72.40 ± 98.45	80.58 ± 109.63	90.04 ± 92.64	$X^2_{KW}(6) = 13.222$, $p = 0.040$
	Vigorous activity (days)	2.65 ± 2.15	2.25 ± 2.20	2.19 ± 2.02	2.22 ± 2.12	1.92 ± 2.01	1.78 ± 1.96	1.70 ± 1.99	$X^2_{KW}(6) = 12.522$, $p = 0.051$
	Vigorous activity (minutes)	68.49 ± 68.84	53.84 ± 51.22	66.51 ± 98.21	63.10 ± 71.47	63.48 ± 90.73	51.54 ± 64.51	51.57 ± 66.29	$X^2_{KW}(6) = 6.898$, $p = 0.330$
	Seated (horas)	1.10 ± 1.16	1.17 ± 1.49	1.28 ± 2.06	1.20 ± 1.15	0.97 ± 1.23	1.04 ± 0.79	1.33 ± 1.71	$X^2_{KW}(6) = 16.691$, $p = 0.010$
	Total (days)	5.98 ± 1.77	5.51 ± 2.39	5.94 ± 1.81	5.70 ± 2.15	5.26 ± 2.58	5.52 ± 2.22	6.04 ± 1.67	$X^2_{KW}(6) = 6.185$, $p = 0.403$
	Total (minutes/week)	177.44 ± 124.60	161.77 ± 130.56	173.92 ± 101.68	193.22 ± 127.94	162.78 ± 125.91	169.02 ± 114.67	187.31 ± 117.32	$X^2_{KW}(6) = 7.622$, $p = 0.267$
Weekly energy expenditure (MET- minutes/week)									
	Walking	998.83 ± 1142.54	934.55 ± 1161.94	806.82 ± 876.36	884.67 ± 958.80	740.60 ± 961.84	809.02 ± 823.87	960.69 ± 792.29	$X^2_{KW}(6) = 16.573$, $p = 0.011$
	Moderate activity	926.51 ± 940.07	890.16 ± 1109.53	844.75 ± 914.12	817.96 ± 1063.24	888.21 ± 1148.72	855.75 ± 1016.64	1136.41 ± 1163.47	$X^2_{KW}(6) = 11.969$, $p = 0.063$
	Vigorous activity	1933.95 ± 2301.28	1484.72 ± 1820.99	1385.42 ± 1631.38	1547.40 ± 2056.57	1357.18 ± 2069.39	1072.81 ± 1510.07	1127.46 ± 1702.63	$X^2_{KW}(6) = 12.042$, $p = 0.061$
	Total	3859.29 ± 3487.25	3309.43 ± 3423.09	3036.98 ± 2389.79	3498.10 ± 3489.97	2985.98 ± 3418.45	2737.58 ± 2624.55	3224.57 ± 2870.32	$X^2_{KW}(6) = 8.358$, $p = 0.213$
Vigorous physical activity criteria									
≥3 days of vigorous physical activity [A]	No	23 (53.5)	39 (63.9)	57 (59.4)	46 (63.0)	108 (69.2)	88 (68.8)	98 (73.1)	$X^2(6) = 9.482$, $p = 0.148$
	Yes	20 (46.5)	22 (36.1)	39 (40.6)	27 (37.0)	48 (30.8)	40 (31.3)	36 (26.9)	
≥7 days of any physical activity [B]	No	22 (51.2)	37 (60.7)	62 (64.6)	40 (54.8)	104 (66.7)	81 (63.3)	81 (60.4)	$X^2(6) = 5.715$, $p = 0.456$
	Yes	21 (48.8)	24 (39.3)	34 (35.4)	33 (45.2)	52 (33.3)	47 (36.7)	53 (39.6)	
≥3 days of vigorous physical activity [C]	No	24 (55.8)	39 (63.9)	56 (58.3)	46 (63.0)	105 (67.3)	88 (68.8)	97 (72.4)	$X^2(6) = 7.872$, $p = 0.248$
	Yes	19 (44.2)	22 (36.1)	40 (41.7)	27 (37.0)	51 (32.7)	40 (31.3)	37 (27.6)	
Categories of physical activity									
≥5 days of moderate physical activity/walking [D]	No	15 (34.9)	23 (37.7)	42 (43.8)	29 (39.7)	77 (49.4)	55 (43.0)	42 (31.3)	$X^2(6) = 11.168$, $p = 0.083$
	Yes	28 (65.1)	38 (62.3)	54 (56.3)	44 (60.3)	79 (50.6)	73 (57.0)	92 (68.7)	
≥5 days of any physical activity [E]	No	10 (23.3)	18 (29.5)	19 (19.8)	18 (24.7)	50 (32.1)	37 (28.9)	30 (22.4)	$X^2(6) = 6.891$, $p = 0.331$
	Yes	33 (76.7)	43 (70.5)	77 (80.2)	55 (75.3)	106 (67.9)	91 (71.1)	104 (77.6)	
Categories of physical activity									
	Light	8 (18.6)	18 (29.5)	19 (19.8)	17 (23.3)	48 (30.8)	37 (28.9)	30 (22.4)	$X^2(12) = 10.892$, $p = 0.547$
	Moderate	11 (25.6)	13 (21.3)	31 (32.3)	18 (24.7)	42 (26.9)	36 (28.1)	44 (32.8)	
	Vigorous	24 (55.8)	30 (49.2)	46 (47.9)	38 (52.1)	66 (42.3)	55 (43.0)	60 (44.8)	
**Health- related quality of life**									
Physical component									
	Physical functioning (%)	96.20 ± 11.33	95.57 ± 10.71	96.01 ± 8.74	93.97 ± 11.06	93.10 ± 9.76	90.99 ± 11.36	87.61 ± 12.98	$X^2_{KW}(6) = 96.472$, $p < 0.001$
	Role-physical (%)	38.60 ± 20.57	30.41 ± 17.97	33.02 ± 17.08	35.27 ± 17.62	34.36 ± 17.53	33.48 ± 16.11	38.69 ± 18.22	$X^2_{KW}(6) = 17.968$, $p = 0.006$
	Bodily pain (%)	81.41 ± 17.57	83.11 ± 18.60	81.58 ± 16.58	78.79 ± 17.88	76.84 ± 19.43	73.51 ± 17.36	70.22 ± 20.73	$X^2_{KW}(6) = 38.067$, $p < 0.001$
	General health (%)	49.79 ± 9.19	48.66 ± 11.04	48.48 ± 10.77	48.88 ± 10.97	49.76 ± 10.45	50.92 ± 9.94	51.85 ± 10.29	$X^2_{KW}(6) = 11.033$, $p = 0.087$
Mental component									
	Vitality (%)	49.32 ± 11.32	51.43 ± 15.31	48.13 ± 13.80	48.92 ± 12.15	48.82 ± 12.81	49.84 ± 13.98	51.24 ± 13.74	$X^2_{KW}(6) = 5.471$, $p = 0.485$
	Social functioning (%)	80.00 ± 16.62	83.77 ± 17.72	85.83 ± 17.09	81.64 ± 18.26	85.06 ± 17.21	84.61 ± 16.36	80.67 ± 19.97	$X^2_{KW}(6) = 8.468$, $p = 0.206$
	Role-emotional (%)	38.29 ± 22.21	33.22 ± 19.83	34.72 ± 19.00	35.53 ± 20.55	35.56 ± 19.27	35.99 ± 19.79	36.52 ± 18.92	$X^2_{KW}(6) = 3.675$, $p = 0.721$
	Mental health (%)	38.76 ± 10.24	39.40 ± 12.15	37.53 ± 11.56	39.63 ± 12.61	40.26 ± 13.18	41.74 ± 13.92	41.94 ± 14.41	$X^2_{KW}(6) = 6.037$, $p = 0.419$

Table S5. Distribution (n (%)) and average values (M ± SD) of PA and HRQoL variables according to the qualitative job performance assessment (institutional) of PO.

Variables		Performance evaluation		Statistic [G]
		Good	Very good	
**Physical activity**				
Time spent per week				
Walking (days)		4.28 ± 2.35	3.94 ± 2.37	U = 5636.5, p = 0.610
Walking (minutes)		50.83 ± 47.19	65.20 ± 85.73	U = 6708.0, p = 0.431
Moderate activity (days)		2.50 ± 2.28	2.95 ± 2.19	U = 6820.5, p = 0.356
Moderate activity (minutes)		58.89 ± 59.30	76.26 ± 89.56	U = 6716.5, p = 0.425
Vigorous activity (days)		1.28 ± 1.81	2.01 ± 2.05	U = 7429.5, p = 0.092
Vigorous activity (minutes)		31.94 ± 58.99	59.52 ± 77.03	**U = 7883.5, p = 0.025**
Seated (horas)		0.92 ± 0.79	1.15 ± 1.44	U = 6737.5, p = 0.415
Total (days)		5.39 ± 2.35	5.67 ± 2.15	U = 6528.0, p = 0.514
Total (minutes/week)		136.67 ± 106.27	175.29 ± 119.88	U = 7197.5, p = 0.172
Weekly energy expenditure (MET- minutes/week)				
Walking		703.08 ± 841.82	857.59 ± 929.81	U = 6476.0, p = 0.616
Moderate activity		811.11 ± 979.10	949.23 ± 1077.10	U = 6637.5, p = 0.486
Vigorous activity		751.11 ± 1491.15	1346.64 ± 1853.57	U = 7631.0, p = 0.054
Total		2265.31 ± 2512.19	3153.46 ± 3074.90	U = 7239.0, p = 0.157
Vigorous physical activity criteria				
≥3 days of vigorous physical activity [A]	No	15 (3.3)	444 (96.7)	$X^2(1)$ = 2.369, p = 0.124
	Yes	3 (1.3)	229 (98.7)	
≥7 days of any physical activity [B]	No	12 (2.8)	415 (97.2)	$X^2(1)$ = 0.186, p = 0.666
	Yes	6 (2.3)	258 (97.7)	
Moderate physical activity criteria				
≥3 days of vigorous physical activity [C]	No	15 (3.3)	440 (96.7)	$X^2(1)$ = 2.513, p = 0.113
	Yes	3 (1.3)	233 (98.7)	
≥5 days of moderate physical activity/walking [D]	No	9 (3.2)	274 (96.8)	$X^2(1)$ = 0.625, p = 0.429
	Yes	9 (2.2)	399 (97.8)	
≥5 days of any physical activity [E]	No	5 (2.7)	177 (97.3)	$X^2(1)$ = 0.020, p = 0.888
	Yes	13 (2.6)	496 (97.4)	
Categories of physical activity				
Light		5 (2.8)	172 (97.2)	$X^2(2)$ = 1.438, p = 0.487
Moderate		7 (3.6)	188 (96.4)	
Vigorous		6 (1.9)	313 (98.1)	
**Health- related quality of life**				
Physical component				
Physical functioning (%)		93.89 ± 5.63	92.52 ± 11.40	U = 6664.0, p = 0.452
Role-physical (%)		34.44 ± 19.39	34.87 ± 17.68	U = 6356.0, p = 0.709
Bodily pain (%)		76.76 ± 14.42	76.64 ± 19.15	U = 6259.5, p = 0.610
General health (%)		50.53 ± 10.39	50.00 ± 10.44	U = 5622.5, p = 0.602
Mental component				
Vitality (%)		51.39 ± 13.63	49.61 ± 13.43	U = 5784.0, p = 0.743
Social functioning (%)		86.67 ± 17.15	83.36 ± 17.80	U = 5375.0, p = 0.399
Role-emotional (%)		35.19 ± 18.41	35.68 ± 19.63	U = 5887.5, p = 0.830
Mental health (%)		39.26 ±14.80	40.27 ± 13.05	U = 6650.5, p = 0.475
Health Change (%)		61.11 ± 14.51	59.02 ± 15.42	U = 5549.5, p = 0.483

Key:

[A], ≥1500 MET- minutes/week

[B], ≥3000 MET- minutes/week

[C], ≥20 minutes/day

[D], ≥30 minutes/day

[E], ≥600 MET- minutes/week

[F], Last qualitative performance assessment

[G], Teste do X^2 or Mann-Whitney U Test
